# Severe acute myopathy following SARS-CoV-2 infection: a case report and review of recent literature

**DOI:** 10.1186/s13395-021-00266-5

**Published:** 2021-04-21

**Authors:** Badrul Islam, Mohiuddin Ahmed, Zhahirul Islam, S. M. Begum

**Affiliations:** 1 Laboratory Sciences and Services Division (LSSD), International Center for Diarrhoeal Disease Research, Bangladesh (icddr,b), Dhaka, Bangladesh; 2 Bangladesh Specialized Hospital, Dhaka, Bangladesh

**Keywords:** Myopathy, Guillain-Barré syndrome, Nerve conduction, Electromyogram, SARS-CoV2

## Abstract

**Background:**

SARS-CoV2 virus could be potentially myopathic. Serum creatinine phosphokinase (CPK) is frequently found elevated in severe SARS-CoV2 infection, which indicates skeletal muscle damage precipitating limb weakness or even ventilatory failure.

**Case presentation:**

We addressed such a patient in his forties presented with features of severe SARS-CoV2 pneumonia and high serum CPK. He developed severe sepsis and acute respiratory distress syndrome (ARDS) and received intravenous high dose corticosteroid and tocilizumab to counter SARS-CoV2 associated cytokine surge. After 10 days of mechanical ventilation (MV), weaning was unsuccessful albeit apparently clear lung fields, having additionally severe and symmetric limb muscle weakness. Ancillary investigations in addition with serum CPK, including electromyogram, muscle biopsy, and muscle magnetic resonance imaging (MRI) suggested acute myopathy possibly due to skeletal myositis.

**Conclusion:**

We wish to stress that myopathogenic medication in SARS-CoV2 pneumonia should be used with caution. Additionally, serum CPK could be a potential marker to predict respiratory failure in SARS-CoV2 pneumonia as skeletal myopathy affecting chest muscles may contribute ventilatory failure on top of oxygenation failure due to SARS-CoV2 pneumonia.

## Background

Swarming spread of SARS-CoV2 pandemic revealed substantial and varied neuromuscular manifestations [1]. Recent data from Wuhan, China, suggest that neurological symptoms/signs are present in over 30% of patients with severe SARS-CoV2 infection [1]. It spans from involvement of the central to the peripheral nervous system manifesting as headache, dizziness, encephalopathy, epileptic seizures, and stroke or muscle weakness. This involvement indicates its affinity toward different levels of nervous system or the effector tissues like skeletal muscle or sensory receptor organs. Neurotropism of this virus is apparent from the evidence of impairment of test and/or smell sensation early in the course of illness, which specifies its affection toward gustatory or olfactory sensory nerve terminals. The virus is also isolated from the cerebrospinal fluid suggesting potential neuroinvasive nature of SARS-CoV2. Involvement of the peripheral nerves was claimed to have association with SARS-CoV2 infection resulting in Guillain-Barré syndrome (GBS). On the other hand, skeletal muscle damage due to SARS-CoV2 infection has been highlighted in several reports documenting nearly 23% of severe infection [1–3]. Importantly, weakness due to severe myopathy can adversely affect the outcome in SARS-CoV2 pneumonia. Recently acute severe myopathic weakness has been documented in six patients in severe SARS-CoV2 pneumonia requiring mechanical ventilation [3], however the mechanism of skeletal muscle damage remained elusive. Our patient may in part address the pathogenesis of myopathy in SARS-CoV2 infection.

## Case presentation

**Fig. 1:**
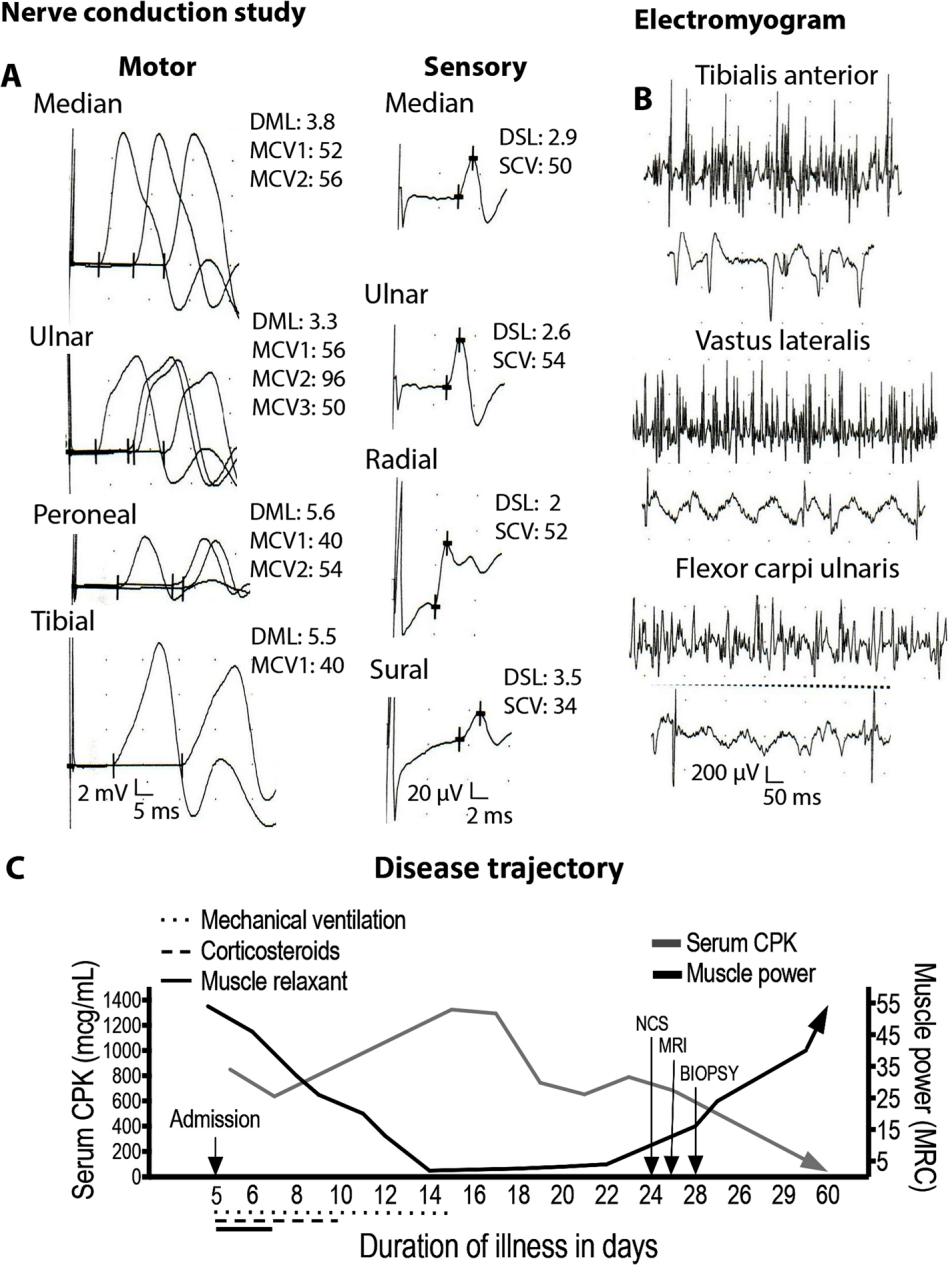
Nerve conduction study, electromyogram, and disease trajectory. (**a**) Motor and sensory nerve conduction was normal despite severe muscle weakness. Compound muscle action potential (CMAP) amplitudes are measured in millivolts (mV); 2 mV per division for all motor study traces. DML, distal motor latency in ms. MCV1, motor conduction velocity in millisecond (ms). MCV2, motor conduction velocity in ms. Sensory nerve action potential (SNAP) amplitudes are measured in microvolts (μV); 20 μV per division for all sensory study traces. DSL, distal sensory latency. SCV, sensory conduction velocity. (**b**) Electromyogram (EMG) showing myopathic motor unit potentials, a full recruitment pattern and spontaneous muscle fiber activity in several sampled muscles. Motor unit potential (MUP) amplitudes are measured in microvolt (μV); 200 μV per division for all EMG traces

**Fig. 2:**
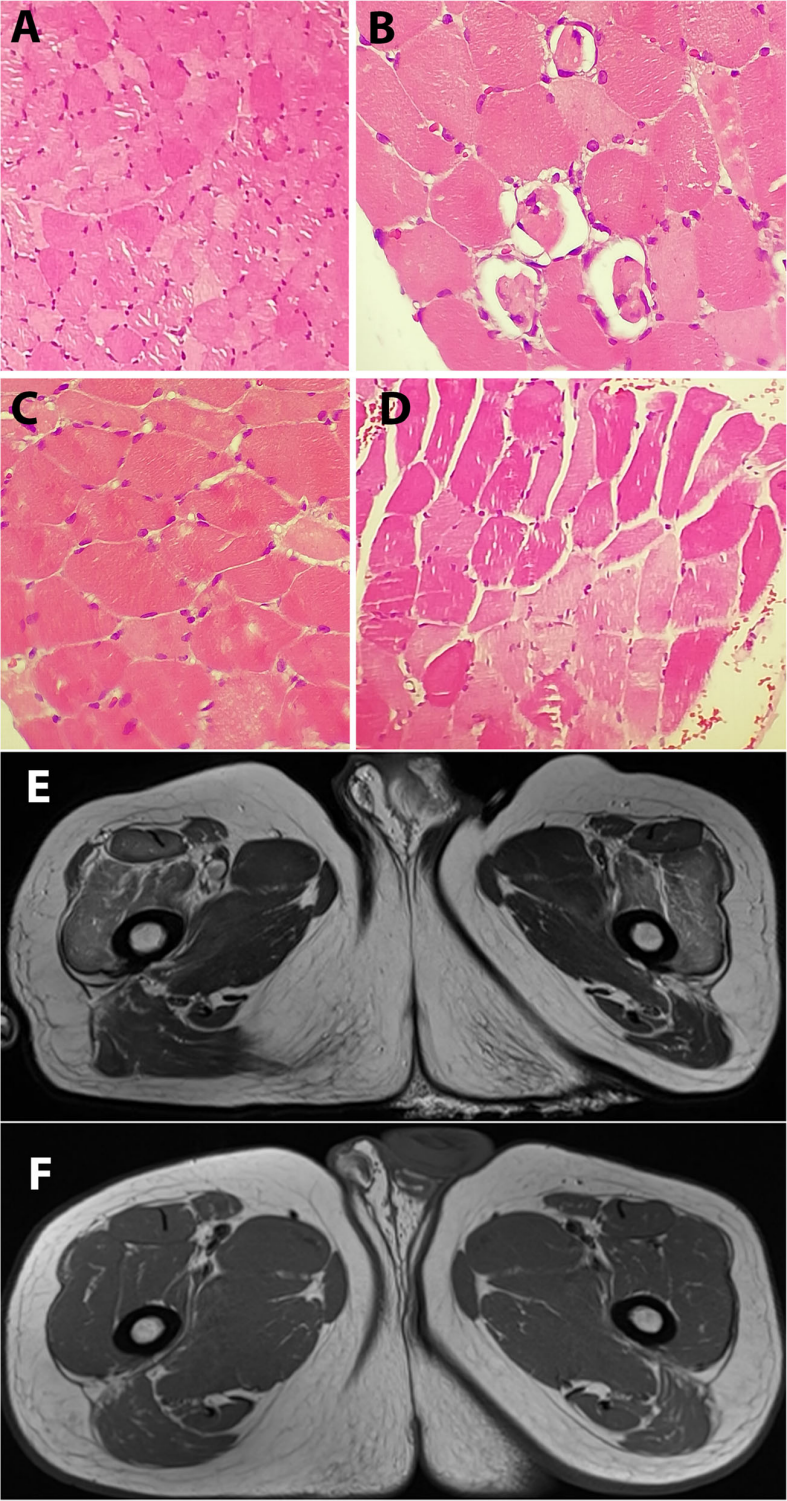
Muscle histopathology and MRI of upper thigh axial sections. (**a**-**d**) Muscle histopathology sections sampled from quadriceps femoris muscle stained with hematoxylin and eosin, showed variation in muscle fiber size with predominantly spherical shape myosites and multifocal and discrete myosite degeneration lacking infiltration of inflammatory cells. (**e**) T2 weighted MRI section of the upper thigh done on day 20, showed marked hyperintensity in both the quadriceps muscles. (**f**) Repeat T2-weighted MRI of the same section of the thigh muscles done after 48 days of the 1st MRI shows both the quadriceps muscles appear normal

A 42-year-hypertensive man having history of bronchial asthma presented with high-grade continuous fever, dry cough, and sore throat. He was brought to hospital for difficulty in breathing, experienced on day 5 of his febrile illness. On admission, he looked toxic, febrile (103 °F) and tachypnoeic (40 breaths/min) with low peripheral SpO2 (85%). He was hypotensive (90/40 mm Hg) having a rapid pulse (120/min). Wheeze and crackles were obvious on chest auscultation especially on mid and lower zones of both lung fields along with woody dull percussion note in the abovementioned areas. This suggested severe SARS-CoV2 pneumonia. He was immediately shifted to intensive care unit (ICU) addressing the severity of chest infection and need for respiratory assistance. High flow oxygen was given through nasal canula, intravenous fluid, and ceftriaxone 1g bd were administered and necessary investigations were sent including deep nasopharyngeal swab for PCR for SARS-CoV2. Inspite of receiving high flow oxygen, due to relentless desaturation of peripheral SpO2, he was orotracheally intubated for assisted mechanical ventilation. His peripheral blood picture showed high total leukocyte count with neutrophilic leukocytosis, elevated serum C-reactive protein, procalcitonin, ferritin, and serum CPK (Table 1). Chest X-ray showed bilateral bronchopneumonic patchy opacities but serum troponin, serum pro-BNP, and echocardiogram was within normal range. He was PCR positive for SARS-CoV2 and received intravenous remdesivir (100 mg daily for 10 days), tocilizumab (8 mg/kg; 2 doses), and dexamethasone (5 mg q6h for 5 days and 5 mg bd for 7 days). Otherwise, in favor, he had intact orientation and his hemoglobin level, coagulation profile (platelet count, PT, and APTT), serum electrolytes, serum anti-GM1 antibody titer and blood sugar level were within normal limits and he had preserved liver and renal functions (Table 1). However, his serum d-dimer was elevated. Subsequently, high resolution spiral CT scan of chest revealed bilateral diffuse ground glass densities and reticulation with features of consolidation and sub-segmental pulmonary embolism. Accordingly, he received subcutaneous low molecular weight heparin for 2 weeks from the second day of his ICU admission. In view of high CRP, increasing O_2_ requirement and inability to sustain a stable blood pressure, tracheal aspirate, blood and urine samples were sent for culture and sensitivity (CS) on the 3rd day of admission to exclude secondary bacterial infection. Ceftriaxone was changed to intravenous teicoplanin (for 10 days) for a broader antibacterial coverage. Tracheal aspirate CS revealed growth of methicillin-resistant Staphylococcus aureus (MRSA), which was fortunately sensitive to teicoplanin. He required sedation (intravenous midazolam and fentanyl for 10 days) and muscle relaxation (IV vecuronium for 2 days) to maximize the tolerability of mechanical ventilation. On the 10th day of his mechanical ventilation, when sedation was gradually withdrawn, weaning from ventilator was unsuccessful and marked symmetrical upper and lower limb weakness was apparent. Both his upper and lower limbs were paralyzed (power 0/5), moderately wasted, flabby, and deep tendon reflexes could not be elicited with an intact sensation in all modalities. However, he was fully alert, and functions of all cranial nerves were preserved. At this time, serum CPK was higher (1325 mcg/mL) than at admission (850 mcg/mL). Electrophysiology done on day 20 of admission revealed normal motor and sensory conduction but myopathic changes on needle electromyogram (EMG) examination, consisting of spontaneous muscle fiber activity, small motor unit potentials, and a full recruitment pattern (Fig. 1). Thigh and calf muscle MRI done on day 20, showed marked hyperintensity in both the quadriceps muscles (Fig. 2e), mild hyperintensity in the hamstrings, and patchy hyperintensity in the leg muscles. Skeletal muscle histopathology done on day 23 and muscle sections sampled from quadriceps femoris showed variation in muscle fiber size with predominantly spherical shape myosites and multifocal and discrete myosite degeneration lacking infiltration of inflammatory cells (Fig. 2a-d). Other specific histopathologic or histochemical analyses could not be done. Eventually, he could be weaned from respirator and maintain SpO2 with ambient air at day 18 from the day of intubation. He was mechanically ventilated with control/assist control mode ventilation for the 1st 2 days and then put onto synchronized intermittent mandatory ventilation (SIMV) mode for 12 days and was on only pressure support ventilation (PSV) for the last 4 days using continuous positive airway pressure (CPAP) mode ventilation.

**Table 1:** Laboratory investigation findings of the presented case with acute myopathy following SARS-CoV2 infection

Investigations (normal value and unit of measurement)	Patient value on admission and range during hospital stay
Hemoglobin (13.5-17.5 g/dl)	14.5 (14.5-9.2)
Total leukocyte count (4500 to 11,000/μl)	21,100 (10,170-31,780)
Polymorph (40-65%)	93% (62-94)
Lymphocyte (30-50%)	4% (4-36)
Total platelet count (150,000 to 400,000/μl)	295,000 (270,000 to 480,000/μl)
C-reactive protein (< 5 mg/L)	300 (2.7-300)
Serum procalcitonin (0.10-0.49 ng/mL)	5.69 (0.27-5.69)
Serum ferritin (< 250 ng/mL)	811 (799-2185)
Serum d-dimer (< 0.4 mcg/mL)	1.25 (1.25-5.55)
Serum PT (11-13.5 s)	13 (13-15)
Serum aPTT (25-35 s)	33 (30-33)
Serum CPK (< 120 mcg/mL)	850 (637-1325)
Serum electrolytes	
Na^+^ (135-145) mmol/L	138 (134-152)
K^+^ (3.5-5.5) mmol/L	4.6 (3.5-5.1)
Ca^+^ (8.5-10.5) mg/dl	8.6 (7.6-8.6)
Mg^+^(1.5-2.5) mmol/L	1.9 (1.9-3)
Serum creatinine (60-110 μmol/L)	87 (71-155)
Serum SGPT (7-56 U/L)	32 (32-45)
Serum troponin-I (< 0.04 ng/ml)	0.02 (0.02-0.2)
Serum Pro-BNP (< 125 pg/mL)	130 (109-130)
Serum anti GM1 antibody	(− ve)
Cerebrospinal fluid (CSF)	
Total protein (up to 45 mg/dl)	10
Total WBC count (0-5/cmm)	05

*PT* prothrombin time, *aPTT* activated partial thromboplastin time, *CPK* creatinine phosphokinase, *SGPT* serum glutamic pyruvic transaminase, *BNP* brain natriuretic peptide, *WBC* white blood cells

He became SARS-CoV2 negative on repeat PCR on day 15 of his onset of weakness. Improvement of his limb muscles showed fair progress (muscle power: distal= 4/5 and proximal= 3/5) when he was discharged from hospital after 4 weeks of his hospital stay. His muscle power was normalized 1 month after his discharge from hospital. At this time, his serum CPK, d-dimer, and serum ferritin were within normal limit and a repeat MRI of his lower limb muscles appeared almost normal (Fig. 2f).

## Discussion and conclusions

Severe acute myopathy can be a life-threatening complication in SARS-CoV2 infection. Our patient presented with SARS-CoV2 pneumonia complicated to acute respiratory distress syndrome (ARDS) and eventually developed features of severe myopathy in quick succession. However, serum CPK was already elevated on admission suggesting the particular potential to damage the skeletal muscle tissue from the beginning of illness. Throughout the illness, except severe pneumonia, sepsis, and skeletal muscle damage, other major organ functions were stable. SARS CoV’s predilection for the skeletal muscle was known from the pandemic that broke in March 2003 [4, 5]. At that time, in addition to lung damage, muscle weakness and elevation of serum CPK level was documented in more than 30% of the SARS-infected patients [6]. Similar myopathogenic potential has also been documented currently in around 20% severe SARS-CoV2 infection [1]. However, the pathogenesis of muscle damage still remains unknown.

A series of six cases with acute myopathy has recently been published from Italy [3]. All the cases in the Italian series showed marked prolongation of motor nerve distal Compound Muscle Action Potential (dCMAP) duration, which was absent in our case. Prolongation of dCMAP indicates distal motor nerve myelin damage, frequently present in demyelinating GBS. Terminal motor axons are vulnerable to immune-mediated attack because here the blood nerve barrier is deficient and axons become unmyelinated just before they join the myoneural junction. Therefore, immune mediated or direct viral invasion at the nerve terminals could be a possibility of paranodal disruption resulting features of distal demyelination. However, evidence goes in favor of indirect muscle injury, as viral particles could not be isolated from damaged skeletal muscle tissues of the patients infected with SARS-CoV [4]. Additionally, prolongation of dCMAP has also been documented in some cases of critical care myopathy, postulated slowing of the muscle fiber conduction velocity, and reduced sarcolemmal excitability [7].

Our patient presented with rapidly progressing symmetrical quadriplagia and loss of deep tendon reflexes. We assumed the possibility of Guillain-Barré syndrome (GBS), which has been documented to be associated with SARS-CoV2 infection in recent literatures [8–10]. Based on our interpretation, this association of GBS and SARS-CoV2 infection could be co-incidental as sufficient epidemiological evidence is lacking which was very obvious between GBS and Zika virus infection in recent past [11]. Our clinical suspicion of GBS was also doubted, as despite having marked muscle weakness, our patient did not have any cranial nerve deficits, elevated serum CPK, and cerebrospinal fluid assessment did not reveal albuminocytological dissociation. Evaluation of serum anti-GM1 antibody was within normal limit, which is a potential marker for pure motor AMAN variant of GBS, prevalent in Bangladesh [12]. Eventually, electrophysiology suggested myopathic involvement. We admit that ARDS, severe sepsis, and potentially myopathogenic medications like corticosteroids and muscle relaxants that had been used in the ICU could possibly trigger critical illness polyneuropathy/myopathy (CIP/CIM) [13] and in part may contribute in the disease process. However, muscle relaxant was used for only 2 days and serum CPK was already high at admission when none of these medications were used. Moreover, in critical care, myopathy raised serum CPK is not common. In light of recent evidence on CIP/CIM animal model, steroid may not be substantially contributing to CIM [14, 15]. MRI of the pelvis and lower limb muscles clearly showed hyperintensity in both quadriceps and leg muscles suggestive of myositis. However, muscle histopathology showed multifocal and discrete myosite degeneration without infiltration of inflammatory cells suggesting noninflammatory pathology. However, role of neucleotide analog antiviral drug remdicivir and anti-IL6 antibody tocilizumab were unlikely to contribute in the process of weakness but they might influence the muscle biopsy findings specially extinguishing inflammatory evidences where corticosteroids could also had a substantial role. These muscle histopathology features are nonspecific and similar to CIM or viral myositis found in influenza B. But infectious nature of myositis was established as influenza B virus could be isolated from skeletal muscle tissue [16]. Importantly, even in the past, neither muscle inflammation nor SARS-CoV could not be isolated from cadaveric skeletal muscle tissue from confirmed SARS-CoV infected patients with features of severe myopathy, suggesting CIM, or indirect myotoxic effect of this virus [4]. This is acknowledged that Th1 cell-mediated cytokines can produce inflammatory response in muscle tissue [17]. Interestingly, cytokine storm in SARS-CoV2 is also Th1 driven, that can induce muscle inflammation [18].

We admit, due to limited laboratory facilities, we could not explore further to identify the cause of this skeletal myopathy. Since thick filament myopathy is the commonest form of CIM, assessment of the content of myosin in relation to actin in the skeletal muscle tissue through electrophoretic separation could help a step forward to ascertain this type of myopathy with more precision.

Although we were not able to determine whether our patient had CIM or SARS-CoV2 associated myopathy, the following arguments are in favor of CIM: (i) an episode of ARDS requiring prolonged mechanical ventilation, (ii) use of myopathogenic medication like muscle relaxants and corticosteroids. On the other hand, SARS-CoV2-associated myopathy is favored by (i) presence of high serum CPK before the onset of mechanical ventilation, (ii) the fact that high serum CPK is unusual in CIM [19, 20], (iii) the remarkable synchronicity of myopathy and SARS-CoV2 infection, (iv) muscle MRI imaging compatible with myositis, (v) the similarity of our case with an Italian series of SARS-CoV2 cases [21], and (vi) epidemiologic evidence of myopathogenicity in SARS-CoV2 infection [1–3].

In conclusion, we want to stress the possible myotoxic effect of SARS-CoV2 that should be carefully assessed particularly in severe SARS-CoV2 infection. In addition, it merits exploring, whether serum CPK is a potential prognostic indicator for muscle weakness and prolonged mechanical ventilation.

## Data Availability

All data generated or analyzed during this study are included in this published article.

## Abbreviations

CPK: Serum creatinine phosphokinase
EMG: Electromyogram
ARDS: Acute respiratory distress syndrome
MV: Mechanical ventilation
MRI: Magnetic resonance imaging
GBS: Guillain-Barré syndrome
AMAN: Acute Motor Axonal Neuropathy
ICU: Intensive care unit
PT: Prothrombin time
aPTT: Activated partial thromboplastin time
SGPT: Serum glutamic pyruvic transaminase
BNP: Brain natriuretic peptide
WBC: White blood cells
CS: Culture and sensitivity
MRSA: Methicillin-resistant Staphylococcus aureus
dCMAP: Distal Compound Muscle Action Potential
CIP: Critical illness polyneuropathy
CIM: Critical illness myopathy
